# Identification, differentiation and antibiotic susceptibility of *Gallibacterium* isolates from diseased poultry

**DOI:** 10.1186/s13620-018-0116-2

**Published:** 2018-02-05

**Authors:** Hosny El-Adawy, Herbert Bocklisch, Heinrich Neubauer, Hafez Mohamed Hafez, Helmut Hotzel

**Affiliations:** 1 Friedrich-Loeffler-Institut (Federal Research Institute for Animal Health), Institute of Bacterial Infections and Zoonoses, Naumburger Str. 96a, 07743 Jena, Germany; 2 Department of Poultry Diseases, Faculty of Veterinary Medicine, Kafrelsheikh University, Kafr El-Sheikh, 35516 Egypt; 3 Bad Langensalza, Germany; 4 Institute for Poultry Diseases, Free University Berlin, Königsweg 63, 14163 Berlin, Germany

**Keywords:** *Gallibacterium*, Poultry, PCR, RFLP, Antibiotic resistance

## Abstract

**Background:**

*Gallibacterium anatis* is an opportunistic pathogen of intensively reared poultry causing oophoritis, salpingitis, peritonitis and enteritis. *Gallibacterium anatis* infection often remains undiagnosed. Recently multi-drug resistant isolates have been described.

**Methods:**

A newly developed PCR restriction fragment length polymorphism assay targeting the 16S rRNA gene was used to identify and differentiate *Gallibacterium* isolates from chicken, turkey and partridge samples originating from 18 different geographical locations in Thuringia, Germany. Antimicrobial susceptibility to 19 compounds of different classes was assessed.

**Results:**

Nineteen *Gallibacterium* isolates were investigated. In 9 birds (47.4%) *Gallibacterium* species were isolated exclusively while in 10 birds (52.6%) other bacterial or viral agents could be detected in addition. In one chicken a mixed infection of *Gallibacterium anatis* and *Gallibacterium* genomospecies was identified. All isolates were susceptible to apramycin, florfenicol and neomycin and resistant to clindamycin, sulfathiazole and penicillin. Resistance to sulfamethoxim, spectinomycin, tylosin and oxytetracycline was observed in 93.3%, 93.3%, 86.7% and 80.0% of the field strains, respectively.

**Conclusions:**

The PCR-RFLP assay allows specific detection and differentiation of *Gallibacterium* spp. from poultry. Antimicrobial resistance of *Gallibacterium* spp. is highly significant in Thuringian field isolates.

## Background

*Gallibacterium* (*G.*) is a genus within the *Pasteurellaceae* family and is associated with a wide spectrum of avian host species based on isolations from domestic and wild birds including chickens, turkeys, geese, ducks, pheasants and partridges [1–4]. Chickens are the preferred host of *Gallibacterium* spp., which constitute a part of the normal flora in the upper respiratory and the lower genital tract [4–6]. *G. anatis* infections in chicken resulted in a variety of signs and lesions such as respiratory problems, necrosis in liver, peritonitis, salpingitis, haemorrhagic and ruptured follicles and a drop in egg production [6–9]. Mixed infections with other poultry pathogens such as *Escherichia* (*E*.) *coli* contributed to the major lesions with *G. anatis* in naturally affected chickens [9].

Genus *Gallibacterium* belongs to phylum Proteobacteria, class Gammaproteobacteria and family *Pasteurellaceae* [10, 11]. *Gallibacterium* was isolated for first time in 1950 from cloaca of healthy chickens and was described as haemolytic “cloaca bacterium” by Kjos-Hansen [12, 13]. Being similar to *Pasteurella* (*P.*) in several characters, it was earlier known as *P. anatis*. The genus name *Gallibacterium* was firstly given by Bisgaard in the year 1982 on the basis of certain phenotypic characters used for identification of isolates of *Actinobacillus salpingitidis* and avian *P. haemolytica* [1, 5, 14, 15]. Genus *Gallibacterium* was established within the family of *Pasteurellaceae* based on 16S rRNA gene sequences [16]. The genus includes strains belonging to *G. anatis*, *G. genomospecies* [3, 5, 10] and unnamed group V [15]. Taxon 1 designated as a third group of strains [14] and named *P. anatis*, which was also found closely related to *A. salpingitidis* and avian *P. haemolytica* [11].

The genus *Gallibacterium* comprises four species, namely *G. anatis*, *G. melopsittaci* sp. nov., *G. trehalosifermentans* sp. nov., and *G. salpingitidis* sp. nov., and three *G*. genomospecies [15, 17].

*G. anatis* can be further sub-divided into two phenotypically distinct biovars: *haemolytica* and the non-haemolytic biovar *anatis* [17]. *G. anatis* isolates were formerly known as strains of the avian *Pasteurella haemolytica–Actinobacillus salpingitidis* complex or *Pasteurella anatis*. Haemolytic *Pasteurella*-like bacteria (*Gallibacterium anatis*) are the causing agent of salpingitis with or without peritonitis but also of septicaemia, pericarditis, hepatitis, respiratory tract lesions and enteritis [18, 19]. *G. anatis* is globally distributed and has been isolated from poultry in some countries within Europe [15, 17, 20].

Various tools have been used for the identification of *Gallibacterium* including cultivation, fluorescence in situ hybridization (FISH), genus-specific PCR, matrix-assisted laser desorption/ionization time-of-flight (MALDI-TOF) mass spectrometry and a *gtx*A gene-based quantitative PCR (qPCR) assay.

Diagnosis of the *Gallibacterium* infection was based on isolation and identification including phenotypic characterization [17]. Phenotypic characterization involves laborious and time-consuming methods, which may also give ambiguous results due to variable outcomes. This in turn leads to difficulty in interpretation of the genus and species designations [1]. Problems during isolation are mainly related to poor growth on artificial media, subsequent overgrowth by other bacteria, and difficulties in phenotypic identification.

Although MALDI Biotyper software is able to recognize clonally related *G. anatis* strains isolated from different poultry flocks as well as from different organs, MALDI-TOF mass spectrometry is not very much practiced for routine diagnostics in veterinary medicine [21, 22].

The *gyr*B gene-based qPCR method is very useful and highly sensitive for *G. anatis* detection in addition to time and cost saving compared to conventional PCR and phenotypic methods [23].

Beta-subunit of DNA-dependent RNA-polymerase (*rpo*B) gene sequence-based classification was used within *Pasteurellaceae* [24] and has been recommended for this group of bacteria, particularly in cases where phenotypic identification was difficult [25]. However, phylogenetic comparison of 16S rRNA gene sequences has recently become a key character for bacterial classification [26]. They might also be used directly for identification, or result in subsequent development of polymerase chain reaction assays targeting specific regions of the aforementioned genes.

Effective means of antibiotic treatment are highly needed. The frequency of treatment failure in flocks infected with *Gallibacterium* seems to be a recurrent problem [27, 28]. Currently, very limited information on antimicrobial susceptibility of *G. anatis* is available leaving little insight into the general level of resistance for this species [27]. Emergence of antimicrobial resistance has been observed among several organisms belonging to the *Pasteurellaceae* family [29]. The resistance phenotypes from *Gallibacterium* field strains and other taxa of *Pasteurellaceae* demonstrated a remarkably high prevalence of multidrug resistance [27, 29–31]. *Gallibacterium* emerged in last few years as multi-drug resistant pathogen causing outbreaks with high mortality not only in poultry but also in other pet birds [12].

The aim of this study was to use a molecular biological identification method allowing specific detection and differentiation of *Gallibacterium* spp. A PCR restriction fragment length polymorphism (PCR-RFLP) assay was established which allowed an easy and reliable detection and differentiation of *Gallibacterium* spp. Moreover, the antibiotic susceptibility profiles of *Gallibacterium* isolates of chickens, turkey and partridge raised in federal state of Thuringia, Germany were determined.

## Methods

### Isolation and characterization of bacterial strains

In this study, 120 tissue samples were collected from poultry suffering from respiratory signs and reproductive disorders as a routine diagnosis in 18 different locations in the state of Thuringia, Germany. The collected samples were investigated specifically for *Gallibacterium* spp. and other bacteria and viruses causing similar symptoms. Bacterial isolates were cultured from several organs of diseased and perished chickens, turkey and partridge. The age of birds, organs of isolation and pathological findings for *Gallibacterium* positive samples were demonstrated in Table 1. Pre-enriched non-selective medium (buffered peptone water; Oxoid, Wesel, Germany) was inoculated with the collected samples and incubated for 24 h at 37 °C under aerobic condition. A loopful of liquid medium was streaked out on 5% Columbia blood agar (Oxoid), Dextrose Starch Agar medium (Oxoid) and MacConkey agar (Oxoid), and then incubated aerobically for 24 h at 37 °C.

**Table 1:** Isolated *Gallibacterium* strains, investigated organs and pathological findings in the hosts

Isolate	Host	Age	Investigated organs	Pathological findings
Ge 002	Chicken	Adult	Lung, ovary, spleen	Swollen kidney, ovarian degeneration, liver congestion, haemorrhagic enteritis
Ge 004	Chicken	Juvenile	Proventriculus, lung	Airsacculitis, ovarian atrophy, enlargement of heart, parenchym congestion, swollen kidneys
Ge 006	Chicken	Adult	Larynx, liver, lung, ovary	Laryngitis, swollen kidney, splenomegaly, polyseroritis
Ge 023	Chicken	Adult	Heart, liver, lung, spleen	Ascites, pulmonary oedema, hydropericarditis, hepatomegaly with perihepatitis, haemorrhagic enteritis
Ge 024	Chicken	Adult	Larynx, ovary	Airsacculitis, splenomegaly, ovarian degeneration
Ge 081	Chicken	Adult	Ovary, spleen	Lung abscess, enteritis, serositis
Ge 082	Chicken	Adult	Ovary, heart, kidney, liver, spleen	Enteritis, pulmonic abscess, serositis
Ge 095	Chicken	Adult	Lung	Nasal sinusitis, pulmonary oedema, swollen liver
Ge 100	Chicken	Juvenile	Lung	Enteritis, hepatitis, hepatomegaly and splenomegaly
Ge 103	Chicken	Juvenile	Lung, spleen	Liver congestion, pulmonary oedema, haemorrhagic enteritis, swollen kidneys, yolk sac persistence
Ge 134	Chicken	Juvenile	Air sac, brain, eye, heart, larynx, lung	Appendicitis, conjunctivitis, hepatitis, pulmonary oedema, rhinitis
Ge 156	Chicken	Adult	Kidney, lung, spleen	Crop inflammation, enteritis, parenchymitis
Ge 168	Chicken	Juvenile	Kidney, lung, trachea	Conjunctivitis, uveitis, airsacculitis and enteritis, laryngitis, pneumonia, hepatomegaly, nephritis, splenomegaly
Ge 173	Chicken	Adult	Heart, lung	Liver paleness
Ge 186	Chicken	Adult	Proventriculus, lung	Air sacculitis, enteritis
Ge 223	Chicken	Adult	Proventriculus, lung	Hepatomegaly, nephritis, enteritis, spleen inflammation
Ge 297	Chicken	Juvenile	Brain, larynx, trachea	Ovarian atrophy, enteritis, laryngitis
Ge 160	Partridge	Juvenile	Brain, lung	Pneumonia, airsacculitis, enteritis, hepatitis, nephritis
Ge 258	Turkey	Juvenile	Nasal cavity	Enteritis, septicaemia, serositis, swollen parenchymes

Phenotypical characterization of isolated strains was performed as previously reported [32, 33]. Suspected bacterial colonies were 0.5–1.5 mm in diameter, bright translucent, low convex and mostly showed beta-haemolysis on blood agar. When observed obliquely with transmitted light these colonies showed concentric rings on Dextrose Starch Agar medium and pink colonies on MacConkey agar. *Gallibacterium* were growing on Columbia agar as fine, circular smooth-edged colonies of grey-white colour with conspicuous beta-haemolysis. After 48 h or sub-cultivation on MacConkey agar, large flat grey colonies with a diameter of 4–5 mm with an amber raised centre were observed. These microorganisms were overlooked during microbiological investigations because initially small *Gallibacterium* colonies were overgrown by other microorganisms. Gram as well as Giemsa staining was used for detection of morphologically characteristic appearance of examined suspected colonies. Gram staining showed Gram-negative, coccoid to pleomorphic rods. In all cases bacteria produced catalase, and the majority of the isolates formed oxidase, phosphatase, nitrate reduction and sugar fermentation with acid production.

Typical or suspected colonies were picked for further biochemical identification using API 20 NE (bioMerieux, Nürtingen, Germany).

The collected specimens were tested for *Escherichia coli*, *Mycoplasma gallisepticum*, *Mycoplasma synoviae*, *Clostridium perfringens* and adenovirus using specific recommended methods for each organism [34].

### DNA extraction

For isolation of chromosomal DNA, bacterial cultures from a plate were re-suspended in 200 μl of phosphate-buffered saline and the isolation procedure was performed with High Pure PCR Template Purification Kit (Roche Diagnostics, Mannheim, Germany) according to the instructions of the manufacturer.

### PCR and DNA sequencing of 16S rRNA genes

16S rRNA genes were partially amplified by using primers 41f and 1066r (Table 2) [35]. After an initial denaturation at 96 °C for 60 s, 35 cycles of denaturation (96 °C for 15 s), primer annealing (55 °C for 60 s) and primer extension (72 °C for 90 s) were carried out and followed by final elongation step at 72 °C for 60 s. PCR was performed using 2 μl chromosomal DNA, 5 μl 10 x *Taq* buffer (Genaxxon bioscience GmbH, Biberach, Germany), 0.2 μl *Taq* DNA polymerase (Genaxxon bioscience GmbH), 2 μl dNTP mix (2 mM each; Carl Roth GmbH, Karlsruhe, Germany) and 1 μl of both primers (10 mM; Jena Bioscience, Jena, Germany) in a volume of 50 μl. PCR products were analysed by gel electrophoresis on 1% agarose gels, stained with ethidium bromide and visualised under UV light. The resulting ca. 1 kb fragment was excised and DNA was extracted using QIAquick Gel Extraction Kit (Qiagen, Hilden, Germany) according to manufacturer’s instruction. DNA sequencing of purified PCR products was carried out by cycle sequencing procedure with the BigDye™ Terminator Cycle Sequencing Ready Reaction Kit (Applied Biosystems, Darmstadt, Germany). 41f, 1066r, Galli-1 and Galli-2 (Table 2) were used as sequencing primers. Nucleotide sequences were determined on an ABI Prism 310 Genetic Analyzer (Applied Biosystems).

**Table 2:** Primers used in this study

Primer	Nucleotide sequence	Aim	Amplicon length
41f	5´-GCT CAG ATT GAA CGC TGG CG-3′	Amplification, sequencing	ca. 1000 bp
1066r	5´-ACA TTT CAC AAC ACG AGC TG-3′		
Galli-1	5´-CAA GCC GAC GAT CTC TAG-3′	Sequencing	
Galli-2	5´-TTC GCA CAT GAG CGT CAG-3′		
Galli-3	5′-ATA GTA TCG AGA GAT GAA AGG GGT GG-3′	Amplification	684–686
Galli-5	5′-TAT CAC GTT TGC TTC GAG AGC C-3´		

### Alignment

Multiple sequence alignments were done using AlignX of Vector NTI Suite 8.0 (Informax Inc., Oxford, UK). Based upon sequence data of investigated isolates, dendrograms were generated for both *G.* species in this study and other related organisms (*P. multocida*, *P. anatis*, *Avibacterium paragallinarum*, *Avibacterium avium*, *G. anatis*, *G.* genomospecies and *Bisgaard * taxa) with cluster tree neighbour-joining analysis using the bioinformatics tools of Geneious R9.0.5.1 analysis.

### Detection and differentiation of *Gallibacterium* species

Primers used for a *Gallibacterium* spp. specific PCR assay were chosen on the basis of an alignment of 16S rRNA genes of related avian *Pasteurellaceae* species. PCR was carried out by using primers Galli-3 and Galli-5 (Table 2) under the following conditions: 3 μl DNA extract, 5 μl 10 x *Taq* buffer (Genaxxon bioscience GmbH), 0.2 μl *Taq* DNA polymerase (5 u; Genaxxon bioscience GmbH), 2 μl dNTP mix (2 mM each; Carl Roth GmbH) and 1 μl of each primer (10 mM), and the volume made up to 50 μl by addition of bi-distilled water. The following temperature-time programme was used for amplification: After an initial denaturation step at 96 °C for 60 s, 35 cycles of denaturation (96 °C for 15 s), primer annealing (66 °C for 60 s) and primer elongation (72 °C for 60 s) were performed. PCR was terminated by an elongation step at 72 °C for 60 s. PCR products were analysed as described above. The high annealing temperature in the PCR process avoided a cross-amplification with DNA of other avian *Pasteurellaceae* species. The resulting amplicons had lengths of approximately 680 bp for *G. anatis* and *G.* genomospecies.

For identification of *G. anatis* and *G*. genomospecies and conformation of affiliation to genus *Gallibacterium* digestion of the PCR amplicon with *Rsa*I restriction enzyme (New England Biolabs, Frankfurt, Germany) was used. For differentiation of *Gallibacterium* into *G. anatis* and *G*. genomospecies, digestion of the PCR products with *Xba*I restriction enzyme (New England Biolabs) was performed. 8 μl of PCR products were digested with 1 μl *Rsa* I or *Xba* I using 2 μl of recommended buffers in separate 20 μl reactions. Restriction reactions were carried out at 37 °C for 2 h. Restriction products were analysed by gel electrophoresis on a 1.5% agarose gel, ethidium bromide staining and visualization under UV light.

### PCR specificity and limit of detection

A total of 30 bacterial strains including 15 *G. anatis* (14 field strains and one reference strain), four *G*. genomospecies, five *Pasteurella multocida*, *Pasteurella anatis*, *Avibacterium avium*, *Avibacterium paragallinarum*, *Avibacterium gallinarum*, *Mannheimia haemolytica* and *Riemerella columbina* were used in this study. The sensitivity of the PCR reaction was determined by analysing serial dilutions (1:10) of genomic DNA.

### Antimicrobial susceptibility testing

The antimicrobial susceptibility of 15 *Gallibacterium* isolates was tested by using the broth dilution method against 19 antibiotics of different classes (Table 3). Four out of 19 *Gallibacterium* isolates could not be re-cultivated after applying phenotypic identification, but DNA was extracted for further identification.

**Table 3:**
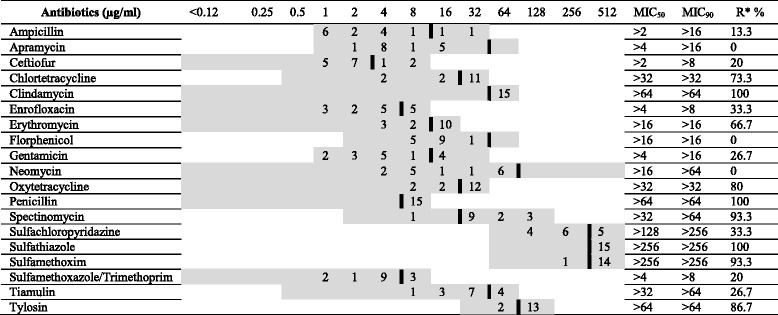
Cut-off values, MIC distribution and MIC_50_/MIC_90_ of 15 *Gallibacterium* isolates

A thick black line indicates the cut-off between clinically sensitive and insusceptible strains

Grey shadow area indicated the test range (μg/ml) of each antimicrobial agent

MIC_50_ = (n × 0.5)

MIC_90_ = (n × 0.9)

^*^R: Resistance rate

Determination of minimum inhibitory concentration (MIC) was performed according to the CLSI standard M31-A2 [36] using a commercially prepared dehydrated panel for *Enterobacteriaceae* (Sensititre-TREK Diagnostic Systems, Cleveland, USA). The plates were incubated for 20–24 h at 35 °C under aerobic conditions. The MIC was defined as the lowest concentration avoiding visible growth. In categorizing the MIC results, CLSI cut-offs for resistance of *P. multocida* were used [36]. Specific cut-offs for respiratory disease agents were used when available. The cut-offs used are indicated in Table 3. *Actinobacillus pleuropneumoniae* ATCC 27090 was included as control.

## Results

### Isolation and phenotypic identification of *Gallibacterium* spp

Nineteen *Gallibacterium* isolates were cultivated and phenotypically identified from 19 diseased or perished birds. The panel of hosts consisted of 17 chickens (*Gallus gallus* f. *domesticus*), one partridge (*Perdix perdix* L.) and one turkey (*Meleagris gallopavo* f. *domesticus*) with different pathological lesions (Table 1), from 18 different farms in Thuringia, Germany. The age of the birds was between 3 weeks and one year. Nine birds were juvenile and 10 of them adult. The reported signs were respiratory symptoms (cold, dyspnea), diarrhoea, anorexia, emaciation of the affected birds and mortality. *Gallibacterium* strains were isolated from lung (14 out of 19), spleen (6/19), ovaries (5/9) and brain (3/19) of diseased birds. Mostly, a septicaemic course of the bacterial infection was diagnosed.

The isolation of *Gallibacterium* in different *Galliformes* species is connected with different pathological findings. The most commonly detected pathological findings were enteritis (11/19) and swollen parenchymes of liver and spleen (10/19). Sometimes, airsacculitis (5/19), ovarian atrophy (4/9) and petichael haemorrhage of different organs (5/19) were observed.

Species identification using the API 20 NE system resulted in classification as *Mannheimia haemolytica* with more than 90%. All isolates showed beta-haemolysis.

In 9 cases only *Gallibacterium* was isolated. In other cases pathogens like mycoplasmas, *Clostridium perfringens*, *E. coli* and adenoviruses were detected besides *Gallibacterium* (Table 4). Other bacterial colonies were collected but could not identified biochemically as *Gallibacterium*.

**Table 4:** Results of species identification via DNA sequencing and PCR-RFLP and occurrence of other pathogens in investigated hosts

Isolates	DNA sequencing	PCR-RFLP results	Other detected pathogens^a^
Ge 002	n. d.^d^	*G. anatis*	*E. coli*
Ge 004	+	*G. anatis*	–
Ge 006	n. d.^d^	*G. anatis*	*E. coli*; adenovirus
Ge 023	+	*G. anatis*	*M. synoviae* ^c^
Ge 024	+	*G. anatis*	–
Ge 081	+	*G.* genomospecies	*E. coli*; adenovirus
Ge 082	+	*G*. genomospecies	*E. coli*; adenovirus
Ge 095	+	*G. anatis*	*E. coli*; *C. perfringens*; adenovirus
Ge 100	+	*G. anatis*	*E. coli*
Ge 103	+	*G. anatis*	–
Ge 134	+	*G. anatis*	adenovirus
Ge 156	+	*G. anatis*	–
Ge 168	+	*G. anatis*	–
Ge 173	+	*G. anatis*	–
Ge 186	n. d.^d^	G. genomospecies/*G. anatis*	–
Ge 223	+	*G. anatis*	–
Ge 297	+	*G. anatis*	*M. gallinarum* ^b^
Ge 160	+	*G. anatis*	–
Ge 258	+	*G. anatis*	*C. perfringens*

^a^Detected by bacteriological, culture or cell culture methods

^b^Detection of antibodies against *M. gallisepticum*

^c^Detection of antibodies against *M. synoviae*

^d^*n. d.* not determined

### Molecular biological identification of *Gallibacterium*

PCR assay based on 16S rRNA genes was established for genus-specific identification of *Gallibacterium* isolates. With primer pair Galli-3 and Galli-5 PCR products of approximately 680 bp were obtained for *G. anatis* and *G*. genomospecies. The high annealing temperature in the PCR process avoided a cross-reaction with DNA of other avian *Pasteurellaceae* species. The specificity of PCR amplification system was tested using DNA of other avian pathogens as PCR templates giving negative results. With this PCR assay it was possible to detect 650 fg/μl of template *Gallibacterium* DNA under described conditions which is equivalent to 880 cfu.

**Fig. 1:**
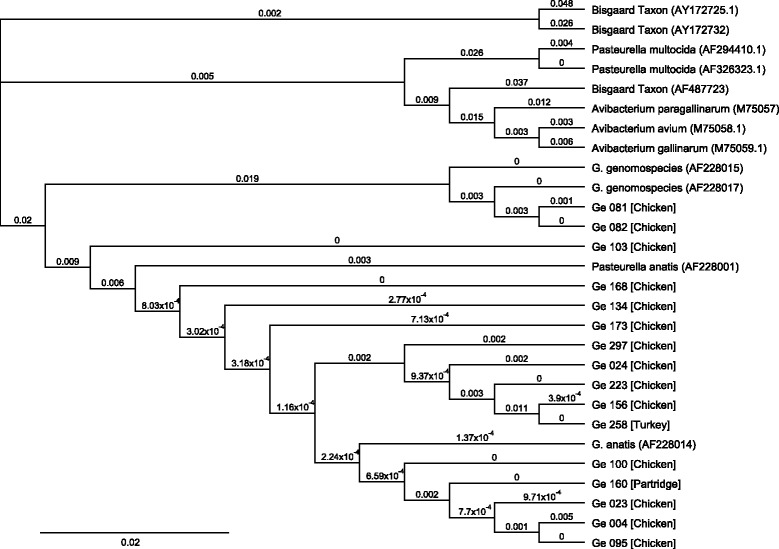
Dendrogram based on sequence profiles of 16S rRNA genes of 16 *Gallibacterium* isolates in this study and related avian *Pasteurellaceae* species and performed with cluster tree neighbour-joining analysis using the bioinformatics tools of Geneious R9.5.0.1 analysis

16S rRNA genes of 16 isolated *Gallibacterium* were partially amplified and sequenced. Species identification was carried out via BLAST search (http://www.ncbi.nlm.nih.gov/BLAST/). The multiple sequence alignments were demonstrated in phylogenetic analysis which showed the relation of isolated *Gallibacterium* from clinical cases and other related organisms. The relatedness of isolated *Gallibacterium* and other avian *Pasteurellaceae* species based on sequence profiles of 16S rRNA gene was demonstrated in Fig. 1.

*Gallibacterium anatis* was identified in 16 birds and *Gallibacterium* genomospecies in 2 chickens (Ge 081 and Ge 082). Both adult hens infected by *G*. genomospecies were kept at the same farm. In one case a mixed culture of *G. anatis* and *G*. genomospecies was detected (Ge 186) (Table 4).

### Differentiation of *Gallibacterium* spp

**Fig. 2:**
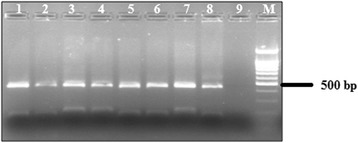
Identification of *G. anatis* and *G*. genomospecies by restriction analysis of PCR products with *Rsa* I; (1 – Ge 002; 2 – Ge 006; 3 – Ge 081; 4 – Ge 082; 5 – Ge 100; 6 – Ge 160; 7 – Ge 186; 8 – Ge 223; 9 – negative control; M – 100 bp DNA ladder)

**Fig. 3:**
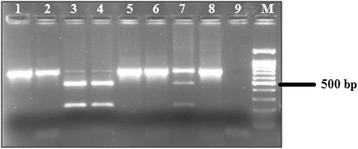
Differentiation between *G. anatis* and *G*. genomospecies by restriction analyses of PCR products with *Xba*I (1 – Ge 002; 2 – Ge 006; 3 – Ge 081; 4 – Ge 082; 5 – Ge 100; 6 – Ge 160; 7 – Ge 186; 8 – Ge 223; 9 – negative control; M– 100 bp DNA ladder)

PCR–RFLP assay based on 16S rRNA genes was established for differentiation of *Gallibacterium* isolates. Use of Galli-3 and Galli-5 primers resulted in approximately 680 bp amplicons. Using *Rsa*I for digestion of amplicons of both species, similar restriction patterns (210 bp and 470 bp) were obtained for *G.* genomospecies and *G. anatis* (Fig. 2). Closely related microorganisms like Bisgaard taxon 40 and 14 have no *Rsa*I restriction site and were not cut (data not shown). Differentiation of *Gallibacterium* isolates into *G. anatis* and *G.* genomospecies was carried out using *Xba*I restriction of PCR products. *G*. genomospecies has an unique restriction site resulting in fragments of 216 bp and 470 bp while *G. anatis* amplicons were not digested (Fig. 3).

### Antimicrobial susceptibility test

The results of antimicrobial susceptibility testing of 15 *Gallibacterium* isolates against 19 antibiotics were demonstrated in Table 3. Four out of 19 *Gallibacterium* isolates could not be re-cultivated after applying identification. All tested isolates were susceptible to apramycin, florphenicol and neomycin, 80.0% of isolates were susceptible to ceftifur and sulfamethoxazole/trimethoprim. 73.3% of isolates were susceptible to gentamicin and tiamulin. Most of isolates showed susceptibility (66.7%) to enrofloxacin and 33.3% were susceptible to erythromycin.

All tested isolates were resistant to clindamycin, sulfathiazole and penicillin. High resistance rates were found to sulfamethoxim and spectinomycin with 93.3%, tylosin with 86.7% and oxytetracycline with 80.0%.

## Discussion

To the best of our knowledge, occurrence of and diseases caused by *Gallibacterium,* species are not well known in Germany. In this study, a total of 19 suspected *Gallibacterium* isolates from different morbid and dead birds were subjected to phenotypic and molecular characterization.

Molecular biological investigation of the isolates resulted in identification of these strains as representatives of the genus *Gallibacterium* within the family *Pasteurellaceae*.

*Gallibacterium* can be isolated from clinically healthy chickens in farms with moderate or low levels of biosecurity [1, 6]. Furthermore, *Gallibacterium* was isolated from birds with various pathological lesions connected with salpingitis, oophoritis, peritonitis, pericarditis, hepatitis, enteritis, upper respiratory tract lesions, and septicaemia [3, 7, 17, 37]. In this study salpingitis, with or without peritonitis, was not detected in any of the cases, but different pathological findings could be found, including enteritis, serositis, hepatitis and nephritis. Various organs of different species were affected. *Gallibacterium* was found as a harmless commensal [3, 5] or in mixed infections with other poultry pathogens such as *E. coli* that shared the major lesions with *G. anatis* in naturally affected chickens [9]. In this study *Gallibacterium* was isolated alone in 9 cases and in 10 cases other pathogens were additionally detected: *E. coli*, *Clostridium perfringens*, *Mycoplasma* species and adenovirus.

The application of MALDI-TOF mass spectrometry and molecular biological methods, especially DNA–DNA hybridization and 16S rRNA sequence analysis, has contributed to an improved *Gallibacterium* classification [17, 21, 22]. A *Gallibacterium* specific PCR assay was used in this study to simplify and shorten accurate identification of suspicious *Gallibacterium* isolates. Molecular biological identification was carried out by sequencing 16S rRNA genes of isolates, and comparison of obtained sequence data with those of GenBank database. Using the described primer system, amplicons were produced which are specific for strains of the genus *Gallibacterium*. No cross-reactivity with closely related bacterial species like Bisgaard taxon 14 and 40 and other members of the *Pasteurellaceae* was recognized. The high annealing temperature in the PCR process avoided a cross-amplification with DNA of other avian *Pasteurellaceae* species. The enzymatic digestion of the amplicons with *Rsa*I was used for confirmation of identified *Gallibacterium* species. Only *Gallibacterium* isolates possess a restriction site for *Rsa*I in the amplified region within members of the *Pasteurellaceae*. This method makes identification of *Gallibacterium* isolates easy. Additionally, an enzymatic digestion using restriction enzyme *Xba*I was suited for species differentiation between species *G. anatis* and *G.* genomospecies.

Antimicrobial susceptibility reports for *Gallibacterium* were relatively rare. In this study, MIC and cut-off from CLSI guidelines and previous reports were used [27, 28]. The number of different techniques and antimicrobial agents made it difficult to compare previous results. Testing of 15 *Gallibacterium* isolates against 19 antibiotics showed that all isolates were susceptible to apramycin, florfenicol and neomycin. 80.0% of isolates were susceptible to ceftiofur and sulfamethoxazole/trimethoprim. 73.3% of isolates were susceptible to gentamicin and tiamulin and most of isolates (66.7%) were sensitive to enrofloxacin. Similar findings were observed in *G. anatis* isolated from both broiler flocks and broiler breeders. They were sensitive to ceftiofur (90.0%), enrofloxacin (93.0%), gentamicin (93.0%), sulfamethoxazole/trimethoprim (83.0%), florfenicol (86.0%), and sulfathiazole (82.0%) [28].

In a previous study, chloramphenicol susceptibility was observed among European strains in Denmark and very high fraction of susceptibilities towards quinolone were detected among 35.0% of Danish *G. anatis* isolates [27]. Moreover, high neomycin susceptibility (64.0%) was observed for Mexican strains isolated from chickens [28]. In contrast to our findings, a higher resistance to neomycin was reported in Taiwan [38]. Only 33.3% of tested isolates were susceptible to erythromycin. In previous studies, it was reported that all of chicken isolates were resistant to erythromycin [38, 39].

Results were in agreement with a study reporting *M. haemolytica* isolated from chicken had high sensitivities to ceftiofur (100%), enrofloxacin (96–100%), florfenicol (92–100%), and sulfamethoxazole/trimethoprim (93–100%) [39]. Similarly, strong sensitivity in *G. anatis* isolates from chickens to ceftiofur and enrofloxacin was reported [38].

Danish and Mexican *Gallibacterium* field strains have high MIC values to a broad range of antimicrobial agents including agents currently used for treatment of *G. anatis* infections. Most prominently, resistance to tetracycline and sulfamethoxazole was observed in 92.0% and 97.0% of field strains, respectively [27]. However, contrasting reports demonstrated moderate to high sensitivity to sulfonamides in isolates originating from broiler flocks and broiler breeders [28].

The resistance rates of isolates to tylosin and oxytetracycline were 86.7% and 80.0%, respectively, which was in agreement with previous study [28] demonstrating that *G. anatis* isolates from poultry revealed resistance to tylosin (100%), clindamycin (97.0%), tetracyclines (80.0–90.0%), and penicillin (70.0%). Similarly, high resistance rates in chickens have been reported for penicillin (60.0–100%) and tetracyclines (72.0–100%) [39]. In contrast moderate sensitivity to tetracycline was reported, too [38].

Most of isolates (86.7%) were resistant to spectinomycin, which was in agreement with a previous study with 0 to 50% spectinomycin sensitive chicken isolates [39]. In another study high sensitivity to spectinomycin was detected in more than 89.0% of the isolates [28].

## Conclusion

The phenotypic identification methods were unable to differentiate the *Gallibacterium* species correctly. The presented study allowed identification and differentiation of *Gallibacterium* isolated from birds by integrated use of phenotypic characterization and subsequent 16S rRNA gene sequence analysis. The description of a PCR-RFLP assay improves the diagnosis and epidemiological understanding of these organisms and allowed correct identification of *G. anatis* and *G.* genomospecies. Future workings will be needed to study the prevalence of *Gallibacterium* species in poultry flocks. This study demonstrates that there is a need for continued monitoring of the antimicrobial susceptibilities of *Gallibacterium* isolates as pathogens in poultry.

## References

[1] Bisgaard M (1993). Ecology and significance of pasteurellaceae in animals. Zentralbl Bakteriol.

[2] Gregersen R, Neubauer C, Christensen H, Korczak B, Bojesen A, Hess M, Bisgaard M (2010). Characterization of *Pasteurellaceae*-like bacteria isolated from clinically affected psittacine birds. J Appl Microbiol.

[3] Mushin R, Weisman Y, Singer N (1980). *Pasteurella haemolytica* found in the respiratory tract of fowl. Avian Dis.

[4] Bojesen A, Nielsen S, Bisgaard M (2003). Prevalence and transmission of haemolytic *Gallibacterium* species in chicken production systems with different biosecurity levels. Avian Pathol..

[5] Bisgaard M (1977). Incidence of *Pasteurella haemolytica* in the respiratory tract of apparently healthy chickens and chickens with infectious bronchitis. Characterisation of 213 strains. Avian Pathol..

[6] Mirle C, Schoengarth M, Meinhart H, Olm U (1991). Studies into incidence of *Pasteurella haemolytica* infections and their relevance to hens, with particular reference to diseases of the egg-laying apparatus. Monatsh Veterinarmed.

[7] Addo P, Mohan K (1985). Atypical *Pasteurella haemolytica* type a from poultry. Avian Dis.

[8] Jordan F, Williams N, Wattret A, Jones T (2005). Observations on salpingitis, peritonitis and salpingoperitonitis in a layer breeder flock. Vet Rec.

[9] Neubauer C, De Souza-Pilz M, Bojesen A, Bisgaard M, Hess M (2009). Tissue distribution of haemolytic *Gallibacterium anatis* isolates in laying birds with reproductive disorders. Avian Pathol..

[10] Christensen H, Bisgaard M, Bojesen A, Mutters R, Olsen J (2003). Genetic relationships among avian isolates classified as *Pasteurella haemolytica*, ‘*Actinobacillus salpingitidis*’ or *Pasteurella anatis* with proposal of *Gallibacterium anatis* gen. Nov., comb. nov. and description of additional genomospecies within *Gallibacterium* gen. Nov. Int J Syst Evol Microbiol.

[11] Mutters R, Ihm P, Pohl S, Frederiksen W, Mannheim W (1985). Reclassification of the genus *Pasteurella* Trevisan 1887 on the basis of deoxyribonucleic acid homology, with proposals for the new species *Pasteurella dagmatis*, *Pasteurella canis*, *Pasteurella stomatis*, *Pasteurella anatis*, and *Pasteurella langaa*. Int J Syst Evol Microbiol.

[12] Singh S, Singh B, Sinha D, Kumar V, Vadhana P, Bhardwaj M, Dubey S. *Gallibacterium anatis*: an emerging pathogen of poultry birds and domiciled birds. J Veterinar Sci Techno. 2016;7(3):1-7.

[13] Kjos-Hansen B (1950). Egglederperitonitt forårsaket av patogen "kloakkbakterie" hos høns. Nord Vet Med..

[14] Bisgaard M (1982). Isolation and characterization of some previously unreported taxa from poultry with phenotypical characters related to *Actinobacillus*-an *Pasteurella* species. Acta Pathol Microbiol Immunol Scand B.

[15] Bisgaard M, Korczak B, Busse H, Kuhnert P, Bojesen A, Christensen H (2009). Classification of the taxon 2 and taxon 3 complex of Bisgaard within *Gallibacterium* and description of *Gallibacterium melopsittaci* sp. nov., *Gallibacterium trehalosifermentans* sp. nov. and *Gallibacterium salpingitidis* sp. nov. Int J Syst Evol Microbiol.

[16] Christensen H, Foster C, Christensen J, Pennycott T, Olsen J, Bisgaard M (2003). Phylogenetic analysis by 16S rDNA gene sequence comparison of avian taxa of Bisgaard and characterization and description of two new taxa of *Pasteurellaceae*. J Appl Microbiol.

[17] Christensen H, Bisgaard M, Bojesen A, Mutters R, Olsen J (2003). Genetic relationships among avian isolates classified as *Pasteurella haemolytica*, ‘*Actinobacillus salpingitidis*’ or *Pasteurella anatis* with proposal of *Gallibacterium anatis* gen. Nov., comb. nov. and description of additional genomospecies within *Gallibacterium* gen. Nov. Int J Sys Evol Microbiol.

[18] Johnson T, Fernandez-Alarcon C, Bojesen A, Nolan L, Trampel D, Seemann T (2011). Complete genome sequence of *Gallibacterium anatis* strain UMN179, isolated from a laying hen with peritonitis. J Bacteriol.

[19] Persson G, Bojesen A (2015). Bacterial determinants of importance in the virulence of *Gallibacterium anatis* in poultry. Vet Res.

[20] Mráz O, Vladík P, Bohácek J (1976). *Actinobacilli* in domestic fowl. Zentralbl Bakteriol Orig A.

[21] Alispahic M, Christensen H, Hess C, Razzazi-Fazeli E, Bisgaard M, Hess M (2011). Identification of *Gallibacterium* species by matrix-assisted laser desorption/ionization time-of-flight mass spectrometry evaluated by multilocus sequence analysis. Int J Med Microbiol.

[22] Alispahic M, Christensen H, Hess C, Razzazi-Fazeli E, Bisgaard M, Hess M (2012). MALDI-TOF mass spectrometry confirms clonal lineages of *Gallibacterium anatis* between chicken flocks. Vet Microbiol.

[23] Wang C, Robles F, Ramirez S, Riber A, Bojesen A (2016). Culture-independent identification and quantification of *Gallibacterium anatis* (*G. anatis*) by real-time quantitative PCR. Avian Pathol.

[24] Korczak B, Christensen H, Emler S, Frey J, Kuhnert P (2004). Phylogeny of the family *Pasteurellaceae* based on *rpo*B sequences. Int J Syst Evol Microbiol.

[25] Christensen H, Kuhnert P, Busse H, Frederiksen W, Bisgaard M (2007). Proposed minimal standards for the description of genera, species and subspecies of the *Pasteurellaceae*. Int J Syst Evol Microbiol.

[26] Ludwig W, Klenk H, Boone D, Castenholz R, Garrity G (2001). Overview: a phylogenetic backbone and taxonomic framework for procaryotic systematics. Bergeys’ manual of systematic bacteriology.

[27] Bojesen A, Vazquez M, Bager R, Ifrah D, Gonzalez C, Aarestrup F. Antimicrobial susceptibility and tetracycline resistance determinant genotyping of *Gallibacterium anatis*. Vet Microbiol. 2011;148(1):105–10. 10.1016/j.vetmic.2010.08.011 20843618

[28] Jones K, Thornton J, Zhang Y, Mauel M. A 5-year retrospective report of *Gallibacterium anatis* and *Pasteurella multocida* isolates from chickens in Mississippi. Poult Sci. 2013;92(12):3166–71. 10.3382/ps.2013-03321 24235226

[29] Aarestrup F, Seyfarth A, Angen Ø. Antimicrobial susceptibility of *Haemophilus parasuis* and *Histophilus somni* from pigs and cattle in Denmark. Vet Microbiolol. 2004;101(2):143–6. 10.1016/j.vetmic.2004.02.012 15172697

[30] Schwarz S, Kehrenberg C, Salmon S, Watts J. *In vitro* activities of spectinomycin and comparator agents against *Pasteurella multocida* and *Mannheimia haemolytica* from respiratory tract infections of cattle. J Antimicrob Chemoth. 2004;53(2):379–82. 10.1093/jac/dkh059 14729757

[31] Kehrenberg C, Walker R, Wu C, Schwarz S. Antimicrobial resistance in members of the family *Pasteurellaceae*. In: Aarestrup F, editor. Antimicrobl resist bacteria of animal origin. Washington, DC: ASM Press; 2006. p. 167–83.

[32] Bisgaard M, Houghton S, Mutters R, Stenzel A. Reclassification of German, British and Dutch isolates of so-called *Pasteurella multocida* obtained from pneumonic calf lungs. Vet Microbiol. 1991;26(1):115–24. 10.1016/0378-1135(91)90048-k 2024434

[33] Dufour-Zavala L, Glisson J, Jackwood M, Pearson J, Reed W, Swayne D, Woolcock P. Isolation, identification and characterization of avian pathogens. In: Am assoc avian Pathol. Volume 5 ed; 2008. p. 12–8.

[34] Swayne D, Glisson J, McDougald L, Nolan L, Suarez D, Nair V. Diseases of poultry. 13th ed. Ames: Wiley-Blackwell; 2013.

[35] Anonymous. Nachweis einer gentechnischen Veränderung von Lactobacillus curvatus in Rohwurst durch Amplifikation der veränderten DNA-Sequenz mit Hilfe der PCR (Polymerase Chain Reaction) und Hybridisierung des PCR-Produktes mit einer DNA-Sonde. Amtliche Sammlung von Untersuchungsverfahren, Deutschland. LMBG. 1997, 35(L 08.00 44).

[36] CLSI. Clinical Laboratory Standards Institute (National Committee for Clinical Laboratory Standards) Performance standards for antimicrobial disk and dilution susceptibility tests for bacteria isolated from animals; 2nd edition: Approved standard. Wayne; 2002;vol. 22 (M31-A2):55-58.

[37] Bisgaard M, Dam A. Salpingitis in poultry. II. Prevalence, bacteriology, and possible pathogenesis in egg-laying chickens. Nord Vet Med. 1981;33(2):81–9. 7232151

[38] Lin M, Lin K, Lan Y, Liaw M, Tung M. Pathogenicity and drug susceptibility of the *Pasteurella anatis* isolated in chickens in Taiwan. Avian Dis. 2001;45(3):655–8. 11569739

[39] Malik Y, Chander Y, Gupta S, Goyal S. A retrospective study on antimicrobial resistance in *Mannheimia* (*Pasteurella*) *haemolytica*, *Escherichia coli*, *Salmonella* species, and *Bordetella avium* from chickens in Minnesota. J Appl Poult Res. 2005;14(3):506–11.

